# Public Psychological Health in COVID-19 Outbreak: Actions and Shortcomings

**DOI:** 10.30476/IJCBNM.2020.86478.1347

**Published:** 2020-10

**Authors:** Azita Jaberi

**Affiliations:** 1 Community Based Psychiatric Care Research Center, Shiraz University of Medical Sciences, Shiraz, Iran

Dear Editor

One of the current worldwide challenges is struggling with the emerging disease of COVID-19. Following the announcement of the outbreak of the disease by the World Health Organization (WHO), numerous measures have been taken in the field of education, treatment and research at the national, regional and international levels by governmental and non-governmental organizations (NGOs).

Iran, like other countries in the world, has taken extensive measures in this regard. On Feb. 25, 2020, the National Headquarter for Combating Corona (NHCC) was formed. ^1^ Immediate and necessary decisions and measures such as closing schools and universities were taken and implemented by this headquarter. The initial instructions about the COVID-19 were published by official websites, including the Ministry of Health and Medical Education (MoHME) and the Universities of Medical Sciences included information on disease symptoms, methods of the prevention, diagnosis, and treatment of COVID-19. 

Another concern about public health was mental and psychological health. Unknown and unpredictable future in the COVID-19 pandemic, along with misinformation and myths, has led to misunderstandings of health messages in the community. Restrictions on travel and quarantine at domestic and international airports in the spring, when most flights take place, have also raised concerns. ^2^

On the other hand, as previous studies have shown, fear of infectious diseases with high prevalence and mortality rate is associated with other psychological challenges such as stigma, discrimination, loss, feelings of fear of getting sick or dying, and helplessness. ^3 , 4^ Therefore, following the activities of the NHCC and MoHME, booklets on stress management and coping with anxiety were published. In this regard, a phone line for psychological counseling has been opened in the Ministry of Health and universities of medical sciences. Meanwhile, children are exposed to a lot of news about the disease epidemic. Therefore, publishing some booklets for children was another measure taken by MoHME to reduce their stress and anxiety.

Another activity that took place in the first weeks of the illness and after the closure of schools and universities in Iran was teaching the students by distant education using virtual content and software. This kind of education ensured the students and their family and made them calm because they did not need to attend schools, universities or dormitories. 

In addition to government agencies, NGOs and charity organizations have taken steps to address the physical, economic, social and psychological harms of the disease. For example, some of these groups provided the equipment and supplies needed for patients and even members of the medical team, such as masks, gloves, and gown. These charity groups have also provided financial support to people suffering from financial problems during the closure of their businesses.

Despite all the measures taken in various areas at the national and provincial levels by government and non-government agencies, there are still some shortages. Because Iran is under numerous sanctions, especially in the field of health, and due to the lack of resources and equipment for healthcare services, an increase in the morbidity and mortality rate might happen in the long time. ^5^

Although some efforts were made regarding the stress and anxiety of the public health, especially children and youth, there are some other measures which could be applied. Through online counseling, psychologists can provide training on how to deal with health issues caused by domestic conflicts, tensions between children and parents, and anxiety about illness. 

It is important to note that financial worries, sanctions, pressures, and deficiencies resulting from them could be associated with physical and mental disorders for people in Iranian society, especially for vulnerable, high risk groups such as low-income individuals and their families. Therefore, the support of international community and WHO, at least in lifesaving medical supplies, is essential. These kinds of lifting sanctions could decrease the tragedy and contribute to preventing the second wave of spreading the disease.
